# Encapsulation of the Antistaphylococcal Endolysin LysRODI in pH-Sensitive Liposomes

**DOI:** 10.3390/antibiotics9050242

**Published:** 2020-05-09

**Authors:** Silvia Portilla, Lucía Fernández, Diana Gutiérrez, Ana Rodríguez, Pilar García

**Affiliations:** 1 Instituto de Productos Lácteos de Asturias (IPLA-CSIC), Paseo Río Linares s/n, Villaviciosa, 33300 Asturias, Spain; qc.silviapv@gmail.com (S.P.); Diana.GutierrezFernandez@ugent.be (D.G.); anarguez@ipla.csic.es (A.R.); pgarcia@ipla.csic.es (P.G.); 2 DairySafe Group, Instituto de Investigación Sanitaria del Principado de Asturias (ISPA), Oviedo, 33011 Asturias, Spain

**Keywords:** endolysins, *Staphylococcus aureus*, liposomes

## Abstract

Phage lysins are promising new therapeutics against multidrug-resistant bacteria. These so-called enzybiotics offer, amongst their most notable advantages, high target specificity and low resistance development. Moreover, there are numerous recent and ongoing studies aimed at demonstrating the efficacy and safety of endolysins in animal models or even in clinical trials. Nonetheless, as is the case for other antimicrobials, it is important to assess potential strategies that may broaden their potential applications or improve their stability. Encapsulation, for instance, has given very good results for some antibiotics. This study sought to evaluate the feasibility of encapsulating an endolysin against the opportunistic human pathogen *Staphylococcus aureus*, one of the most problematic bacteria in the context of the current antibiotic resistance crisis. Endolysin LysRODI has antimicrobial activity against many *S. aureus* strains from different sources, including methicillin-resistant *S. aureus* (MRSA) isolates. Here, this protein was encapsulated in pH-sensitive liposomes with an efficacy of approximately 47%, retaining its activity after being released from the nanocapsules. Additionally, the encapsulated endolysin effectively reduced *S. aureus* cell counts by > 2log units in both planktonic cultures and biofilms upon incubation at pH 5. These results demonstrate the viability of LysRODI encapsulation in liposomes for its targeted delivery under mild acidic conditions.

## 1. Introduction

*Staphylococcus aureus* is a commensal bacterium in the human skin and mucous membranes. However, this microbe is also a leading cause of severe infections for immunocompromised individuals, including skin and soft tissue infections (SSTIs) [1], which are often difficult to cure. Indeed, the increasing prevalence of infections caused by methicillin-resistant *S. aureus* (MRSA) strains results in high rates of treatment failure and relapse. On top of that, the last-resort drug vancomycin has important limitations, such as the potential emergence of resistant strains and its renal toxicity. 

Phage-encoded endolysins specifically degrade the peptidoglycan of the bacterial cell wall. Therefore, they can be used exogenously as antimicrobials (also named enzybiotics), especially against Gram-positive bacteria [2]. These proteins have some valuable advantages compared with antibiotics, such as their narrow spectrum, which prevents altering the beneficial microbiota, and the low rates of resistance development in bacteria after continuous exposure [3]. Moreover, their modular structure facilitates the design of tailored proteins by domain deletion and shuffling [4]. There have been notable efforts aimed at the development of phage-derived lytic proteins against *S. aureus* [5]. Many of these proteins, including CHAP_K_, LysH5, CHAP-SH3b, ClyH, or ClyF, have been shown to help prevent or eliminate biofilms [5]. Also of note, antistaphylococcal endolysins have already been assessed as therapeutics in animal models of infection, and several clinical trials are currently ongoing, such as the ones with proteins SAL200 and CF-301 [5]. Additionally, the commercial product Staphefekt SA.100, which contains a recombinant phage endolysin, is effective for the topical treatment of chronic *S. aureus*-related dermatoses [6,7]. In this context, liposomes are considered to be useful drug delivery vehicles as they improve the therapeutic effect of topical treatments, thereby helping to avoid the serious side effects derived from systemic applications [8]. As a result, liposome encapsulation of phage lysins might enhance their action in skin applications. More generally, the use of nanotechnology for the delivery of antimicrobials opens new possibilities for targeted release of the compound when and/or where it is needed [9]. Thus far, there have been a few examples of encapsulated phage lysins. For instance, Hathaway et al. [10] demonstrated the efficacy of encapsulating a combination of the endolysin domain CHAP_K_ and the bacteriocin lysostaphin in temperature-sensitive liposomes against *S. aureus*. Another study demonstrated that liposome-mediated delivery of lytic proteins was able to successfully kill Gram-negative bacteria without previous permeabilization of the outer membrane [11].

Endolysin LysRODI, encoded by myophage vB_SauM_phiIPLA-RODI [12], exhibits lytic activity against *S. aureus* planktonic cultures and biofilms, and does not select resistant mutants after bacterial exposure to sub-lethal concentrations. Additionally, LysRODI was not toxic in an acute toxicity test performed in zebrafish embryos challenged with the minimum inhibitory concentration (1×MIC) of the protein, which resulted in a high survival rate (>92%). Likewise, it conferred protection against *S. aureus* in a mice model of mastitis [13]. Here, we assessed the encapsulation of LysRODI in pH-sensitive liposomes as a potential strategy to control its delivery under acidic conditions. 

## 2. Materials and Methods

### 2.1. Bacterial Strains and Culture Conditions

The *S. aureus* strains used in this study included Sa9 [14], V329 [15], and 15981 [16]. These strains were routinely grown on Baird-Parker agar (AppliChem, Darmstadt, Germany) plates or in TSB (tryptic soy broth; Scharlau, Barcelona, Spain) at 37 °C with shaking. TSB medium was adjusted at pH 5 with 1 N HCl when necessary. Protein expression was carried out using *Escherichia coli* BL21 (DE3) (EMD Biosciences, San Diego, CA), which was routinely grown in Luria broth (LB) at 37 °C with shaking. When required, ampicillin (Sigma-Aldrich, Madrid, Spain) and IPTG (isopropyl-ß-D-thiogalactopyranoside; Sigma-Aldrich, Madrid, Spain) were added to LB at 100 μg/mL and 1 mM, respectively. 

### 2.2. Protein Purification and Encapsulation

Endolysin LysRODI was purified as described previously [13] and then quantified by using the Quick Start Bradford Protein Assay kit (Bio-Rad, Madrid, Spain). The obtained protein was visualized by SDS-PAGE. The enzymatic activity of the purified endolysin was evaluated against *S. aureus* Sa9 cells using the turbidity reduction assay as described by Obeso et al. [17]. The specific lytic activity was expressed in ∆OD_600_ × min^−1^ × µg^−1^. 

The protein was then encapsulated into pH-sensitive liposomes that release their content at pH values below 5.5. To do that, 2 mL of 0.1 M phosphate-buffered saline (PBS) were added to the formulation named PRONANOSOMES–pH (Nanovex Biotechnologies S.L., Spain), mixed with 135 mM NaCl containing 100 µg/mL of LysRODI, and stored overnight at 4 °C. Then, the sample was heated at 55 °C- 65 °C for 20 min and shaken vigorously for 2 min. After two additional cycles of heating (5 min) and shaking (2 min), the liposomes (vesicle size 450 nm) were purified by ultracentrifugation (65,000 × *g*, 50 min, 4 °C) to remove the unencapsulated protein. To determine the encapsulation efficiency (EE), the generated liposomes were resuspended in PBS (pH 5; 138 mM NaCl, 30 mM KCl, 162 mM Na_2_HPO_4_, 30 mM KH_2_PO_4_), vortexed vigorously, and subjected to ultracentrifugation (65,000× *g*, 5 min, 4°C) to release the endolysin. Protein quantification of the free and encapsulated endolysin was performed by using the Quick Start™ Bradford Protein Assay kit. The encapsulation efficiency (%) was calculated according to the following formula:

$$\mathrm{EE}\;\left(\%\right)=\left(\frac{\mathrm{Cencapsulated}}{\mathrm{Cfree}}+\mathrm{Cencapsulated}\right)\times 100$$

where Cencapsulated and Cfree correspond to the concentrations of encapsulated and non-encapsulated protein, respectively.

Samples from the unencapsulated and encapsulated fraction (the latter after release of the liposomal contents) were also used to perform a turbidity assay in order to confirm that the protein was still active after the encapsulation process. This assay was carried out as described previously [17].

### 2.3. Time-Kill Curves

First, the encapsulated endolysin was released from the liposomes as described above. Then, *S. aureus* Sa9 cultures (OD_600_ ≈ 0.5) were diluted in PBS (pH 5.0; 10^5^ CFU/mL) and challenged with LysRODI released from liposomes or with free endolysin (20 µg/mL, final concentration) as a control. Samples were incubated at 37°C with shaking for 60 min, and viable cell counts were determined at different time points during the incubation (5, 15, 30, 45, 60 min) by performing 10-fold serial dilutions and then spreading them on Baird-Parker (BP) agar plates.

### 2.4. Treatment of Actively Growing Bacterial Cultures and Biofilms

*S. aureus* Sa9 cultures were grown in TSB (pH 5.0) until they reached an OD_600_ of approximately 0.5. Aliquots of these cultures were diluted in fresh TSB (pH 5.0) (10^5^ CFU/mL), mixed with liposomes contaning LysRODI (final protein concentration in the suspension of 4 µg/mL), and incubated at 37 °C with shaking for 4 h. Control samples were treated with buffer, empty liposomes, or 4 µg/mL of native LysRODI.

Biofilms of the different *S. aureus* strains were formed at 37°C in polystyrene 96-well microplates (TC Microwell 96U w/lid nunclon D SI plates, Thermo Scientific, NUNC, Madrid, Spain). To do this, each well was inoculated with 200 μL of a 1:100 dilution from an overnight culture. Following 24 h of incubation under static conditions, the planktonic phase was removed from all wells. Then, wells were washed twice with PBS (pH 7.0), and biofilms were treated with empty liposomes, liposomes containing LysRODI (final protein concentration in the suspension of 40 µg/mL), or free LysRODI (40 µg/mL) in PBS (pH 5.0), and subsequently incubated for 90 min at 37°C. Biofilms were washed twice with PBS (pH 7.0), and the adhered cells were collected by scratching the bottom of the well, and plated on BP agar [12].

### 2.5. Statistical Analysis

Data corresponding to three independent replicates were analyzed with Student’s *t*-test. *p*-values < 0.05 were considered significant.

## 3. Results and Discussion

### 3.1. Protein Encapsulation and Efficacy in Time-Kill Curve Assay

Purified LysRODI endolysin, with a specific activity of 45.00 ± 0.01 (expressed as ∆OD_600_ × min^−1^ × µg^−1^), was subjected to the encapsulation process to obtain pH-sensitive liposomes. The encapsulation efficiency was 46.81%. This result is quite similar to the 49.7% obtained by Hathaway et al. [10] for the encapsulation of CHAP_K_ combined with lysostaphin, and greater than the 35.27% efficiency reported for the encapsulation of endolysin BSP16Lys [11]. 

**Figure 1:**
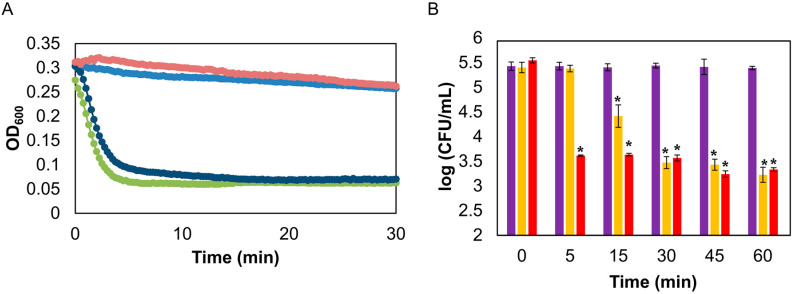
Lytic activity of LysRODI released from pH-sensitive liposomes against *S. aureus* Sa9 in PBS, pH 5. (A) Turbidity reduction assay. Light blue: control without treatment; dark blue: supernatant containing non-encapsulated LysRODI; green: LysRODI released from liposomes; pink: released contents of empty liposomes. (B) Time-kill curve. Purple bar: untreated control culture; yellow bar: culture treated with encapsulated LysRODI (previously released); red bar: culture treated with free LysRODI. *, *p*-value < 0.05.

A turbidity assay was then performed to confirm that the liposome-released endolysin retained its enzymatic activity. The results showed that the protein present in both the non-encapsulated fraction and inside the liposomes were able to efficiently lyse *S. aureus* Sa9 cells (Figure 1A). In contrast, the released contents of empty liposomes did not affect the turbidity of the cell suspension more than the control containing buffer alone (Figure 1A). This indicates that potential remainings of the compounds used for liposome preparation do not have a lytic effect on *S. aureus* cell suspensions.

A time-kill curve was also carried out to determine the antimicrobial activity of the encapsulated protein on exponentially-growing *S. aureus* Sa9 cells. Reductions of about 0.77 ± 0.04 log units and 2.06 ± 0.03 log units were respectively obtained after 15 min and 60 min of incubation when using liposome-released endolysin (Figure 1B). Treatment with the native endolysin showed a significantly higher reduction in viable cell counts than the released endolysin during the first 15 min of incubation. However, after that time point, the bactericidal activity of both protein preparations was very similar. Therefore, it appears that both the encapsulated and the free protein can attain the same level of killing, although the endolysin released from liposomes took slightly longer to start lysing the bacterial cells. The two endolysin encapsulation studies available to date did not compare encapsulated versus free protein. As a result, we do not have information on whether this delay in the start of cell lysis is common in encapsulated lysins. It is difficult to fully determine the cause of this phenomenon, but we could speculate that interactions with the compounds present in the liposomes might slow down LysRODI activity.

### 3.2. Lytic Activity in Actively Growing Cultures

**Figure 2:**
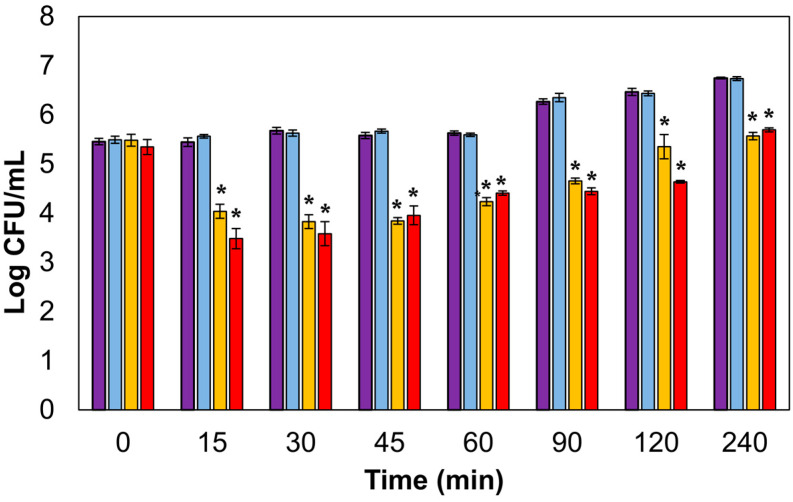
Lytic activity of liposome-encapsulated LysRODI against *S. aureus* Sa9 in tryptic soy broth (TSB; pH 5). Purple bar: untreated control culture; light blue bar: culture treated with empty liposomes; yellow bar: culture treated with encapsulated LysRODI; red bar: culture treated with free LysRODI. *, *p*-value < 0.05.

The antimicrobial potential of liposomes containing LysRODI was also evaluated against *S. aureus* Sa9 cells exponentially growing in TSB medium adjusted at a pH of 5.0. Viable cell count analysis revealed that the greatest level of killing was achieved after 30 min of treatment (Figure 2). At this time point, samples treated with encapsulated endolysin exhibited a reduction of 1.85 ± 0.072 log units compared to the control samples containing empty liposomes. This decrease was similar to that observed when cells were challenged with free LysRODI (Figure 2). After 30 min, cultures treated with either the encapsulated endolysin or the free protein began to grow again as the bacterial growth rate started to outdo the rate of cell lysis. Nonetheless, they did not reach the cell number observed in the controls without LysRODI, even after 4 h of incubation. It must be noted that the time needed for the liposomes to release their contents did not result in a significant delay in the killing dynamics compared to the readily available free endolysin. 

Overall, it seems that endolysin encapsulation in pH-sensitive liposomes could be an effective strategy for controlling growth of contaminating *S. aureus* in mildly acidic environments. Controlled release of phage lytic proteins had not been described so far, the only exception being CHAP_K_, which was successfully delivered using the thermoresponsive nature of the Poly(N-isopropylacrylamide) (PNIPAM) polymer in combination with lysostaphin [10]. Therefore, our results confirm the viability of encapsulating antistaphylococcal phage lysins and broaden the arsenal of possibilities f.or their controlled release.

### 3.3. Antibiofilm Activity of Encapsulated LysRODI

**Figure 3:**
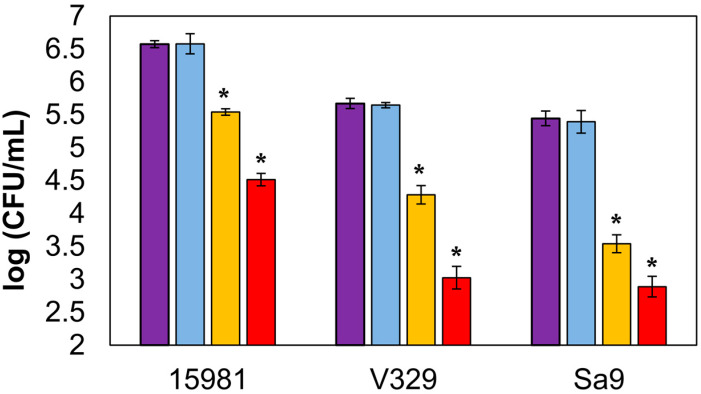
Lytic activity of liposome-encapsulated LysRODI against biofilms formed by *S. aureus* 15981 (polysaccharide biofilm former strain), *S. aureus* V329 (protein biofilm former strain), and *S. aureus* Sa9 (poor polysaccharide biofilm former). Purple bar: untreated control biofilm; light blue bar: biofilm treated with empty liposomes; yellow bar: biofilm treated with encapsulated LysRODI; red bar: biofilm treated with free LysRODI. *, *p*-value < 0.05.

Finally, the activity of encapsulated endolysin was also tested against sessile bacteria. Biofilm treatment with free endolysin led to reductions in viable cell counts of 2.06 ± 0.04, 2.65 ± 0.09, and 2.55 ± 0.05 log units for strains *S. aureus* 15981, *S. aureus* V329, and *S. aureus* Sa9, respectively (Figure 3). In wells treated with encapsulated LysRODI, viable cell counts in the aforementioned strains were reduced by 1.03 ± 0.00, 1.39 ± 0.06, and 1.90 ± 0.03 log units, respectively. Although these reductions were slightly lower than those obtained with the free protein, they still represented a significant decrease in viable biofilm cells. Moreover, we observed antibiofilm activity against all strains, regardless of their biofilm forming ability or the composition of their extracellular matrix. Indeed, the reduction in *S. aureus* V329 (protein matrix) was similar to that observed in strains with a polysaccharidic matrix (*S. aureus* 15981 and *S. aureus* Sa9). However, the bacterial viable reduction was more noticeable in the presence of the free endolysin. To the best of our knowledge, this is the first work that assesses the antibiofilm activity of encapsulated lytic proteins. Moreover, as we mentioned earlier, there are no data comparing the efficacy of encapsulated and free endolysins. For all these reasons, we cannot establish whether encapsulation of these enzymes hampers their ability to move within the biofilm matrix and reach their target cells. A possible explanation might be that the liposomes interact with, and perhaps bind to, the matrix components, somehow preventing them from releasing their contents. In this scenario, part of the encapsulated protein would remain inside the liposomes and never reach their target. Perhaps this would explain why the difference in efficacy between free and encapsulated protein is smaller for the poor biofilm former Sa9, which does not produce a strong extracellular matrix. In that sense, it would be interesting if further work elucidates whether this is a frequent problem with encapsulated lytic proteins or if it is more specific to the type of liposomes chosen for this study. Liposome encapsulation of other types of drugs has given varied results for antibiofilm treatment. For example, some antibiotics seem to benefit from liposome-mediated delivery into the biofilm structure, such as vancomycin, whereas others did not show good results, such as gentamicin [18]. However, there is evidence that the type of liposomes used also has an impact on their activity [19].

## 4. Conclusions

The use of liposome encapsulation opens new possibilities for the delivery of antimicrobials. Indeed, these nanovesicles are thought to enhance the bioavailability, biocompatibility, and safety of the encapsulated compound [20]. The study of endolysins, including the design of novel strategies to deliver them to their target bacteria, is undoubtedly a field of growing interest. This is especially the case due to their low tendency to select resistant mutants, in clear contrast with most other antimicrobials currently in use. Therefore, the potential use of liposome encapsulation for endolysins in order to broaden their applications is a very attractive option. For instance, liposomes have been useful to effectively lyse two Gram-negative bacteria (*Salmonella* Typhimurium and *E. coli*) with endolysin BSP16Lys without pretreatment with an outer membrane permeabilizer [16]. Moreover, by varying the composition of liposomes, it is possible to control the release of the encapsulated lytic protein in response to different environmental cues [21]. An example of this is the temperature-sensitive liposomes used for the release of endolysin CHAP_K_ and lysostaphin at 37 °C (temperature found in wounds), but not at 32 °C (temperature of healthy skin) [10]. Here, we also showed that pH-sensitive liposomes can work for the delivery of endolysin LysRODI to decimate both planktonic and biofilm populations of *S. aureus* in mildly acidic environments. This would include different parts of the human body, such as the skin or the vagina, as well as certain foods or surfaces where staphylococcal contamination might be problematic. It is also worth noting that biofilm formation in *S. aureus* is enhanced at environmental pH values between 4.75 and 5.5, which is precisely within the range of content release for the liposomes used in this work [22]. Nevertheless, although the encapsulated LysRODI was effective against biofilm cells, its activity was significantly hindered by the presence of a thick matrix compared to the free endolysin. Subsequent studies should endeavor to understand the underlying causes of this problem and try to overcome it by altering the composition of the nanovesicles or attempting a combination strategy with matrix-degrading enzymes. Another interesting strategy to explore would be the incorporation of antibodies or other proteins in the liposome surface that can interact with biofilm molecules, thereby facilitating the penetration of the nanovesicle into these sessile structures. Finally, the efficacy of encapsulated endolysins under ex vivo or in vivo conditions remains to be examined, although studies carried out with other antimicrobials have shown promising results. Recently, Hajiahmadi et al. [23] published a highly sophisticated strategy to directly apply encapsulated drugs on skin wounds of MRSA-infected mice. In this study, vancomycin-loaded nanoliposomes coupled with the anti-staphylococcal protein lysostaphin, which acts as a nano-vehicle, were applied. These liposomes specifically bind to the *S. aureus* cell wall using lysostaphin molecules and then disrupt peptidoglycan while releasing the antibiotic. Thus, these liposomes were more effective in suppressing the bacterial infection compared to equivalent doses of untargeted vancomycin liposomes.

Overall, the data presented in this study contribute to our knowledge of the pros and cons of endolysin encapsulation in different types of liposomes. Even though we found that pH-sensitive liposomes may not be ideal for all applications, most notably biofilm elimination, they do offer a new possible option for controlled release of endolysins.
